# Porcelain Facings for Carious Teeth

**Published:** 1884-08

**Authors:** E. C. Moore


### PORCELAIN FACINGS FOR CARIOUS TEETH.

BY E. C. MOORE, D. D. S.

Read Before the Detroit Dental Society.

I will describe to you an operation that, owing either to the lack of appreciation on the part of the patient or the want of patience in the operator, is not as common as in my opinion it should be. The patient had for some years worn an ordinary gold filling upon the labial surface of a central incisor, and during this time had never enjoyed a hearty laugh because of the ever present consciousness of that gold filling, which blazed like the head-light of a locomotive. You may readily appreciate how quickly she grasped at my proposition for her relief.

The defective filling was removed, and the cavity carefully cut into regular shape, the edges being made as straight as possible, and the floor left as nearly level as was practicable, while the edges were given a slight undercut with a sharp engine-bur revolved at great speed ; this part of the operation was not particularly painful. The rubber-dam being still in position, a piece of sheet-wax was rolled between the fingers until it was no larger than a moderate sized needle, and this was molded into the undercut until the walls of the cavity were at right angles with the floor, and the undercut was entirely obliterated. An accurate impression was now taken with plaster-of-Paris, and the patient dismissed. From the impression a model was made and thoroughly dried. A suitable piece of porcelain from a broken artificial tooth had previously been selected, and the color matched to the natural tooth. The side of this which was to lie in contact with the bottom of the cavity was now ground flat upon a coarse stone, and left about the requisite thickness, allowing for the final grinding and polishing. The porcelain piece was now heated and cemented to the end of a piece of orange wood, with a little shellac, the ground side outward for convenience in handling. It was now ground to fit the model as carefully as possible, and the exposed surface polished. The edges were ground to an angle a little less acute than those of the cavity, and were carefully polished with fine stone.

The patient presenting herself, the piece was now fastened in place with Weston's cement, and this is really the most delicate part of the whole operation. The space around the porcelain piece must be sufficient, and yet not too much. The piece must be evenly placed in the cavity, for if there be too much space upon one side there will not be enough on the other for the gold that is to hold it in place. It must be very quickly adjusted, for if this be not the case the cement begins to set and does not hold the piece firmly. The centering of the piece is best secured by inserting the edge of an instrument of a suitable thickness betweeen the porcelain and the tooth at three or four different points.

When the cement was quite hard, a small cavity, equal to what is usually denominated a retaining point, was excavated in the cement and filled with gold. A similar point was then excavated and filled upon the opposite side, and then points midway between these. When enough of these points were excavated and filled, the piece was thus secured so that the remainder of the cement about the edges could be removed and the space filled with gold. The whole was then ground down and polished, when I had a central piece of porcelain surrounded with a delicate ring of gold, which was not unsightly.

I have endeavored to be exact in my description, and perhaps I have made myself tedious. The operation scarcely takes more time in its performance than in its description, but it requires some delicacy of touch and care in its manipulation. There are two or three suggestions that I might make for your benefit. One is to warn you against making the space between the porcelain and the tooth so narrow as to leave insufficient space for enough of gold to securely fasten it. Another is to caution you against excavating the cavity too deeply, and a third is to advise you to postpone to another day the final insertion of the gold, thus allowing plenty of time for the cement to harden.

